# Experiences of Patients Living with Complications of Noncommunicable Diseases  in Mzuzu, Malawi

**DOI:** 10.1002/puh2.70402

**Published:** 2026-10-03

**Authors:** Mary Kumwanje, Thokozani Mzumara, Isaac Ndemera

**Affiliations:** ^1^ University of Livingstonia Mzuzu Malawi; ^2^ Optometry Unit Department of Curative and Rehabilitative Services Mzuzu Urban Community Hospital Mzuzu Malawi; ^3^ Luke International Mzuzu Malawi

**Keywords:** complications, Malawi, noncommunicable diseases, patients

## Abstract

**Background:**

Chronic conditions, such as NonCommunicable Diseases (NCDs) and impairments in body structures, may limit activities of daily living. The quality of care of NCD patients in Malawi is low, leading to a high prevalence of complications of NCD in Malawi.

**Aim:**

The study aimed at exploring the experiences of NCD patients living with complications in Mzuzu, Malawi.

**Methods:**

This qualitative study recruited nine NCD patients with complications. The participants were selected purposively, and where applicable, we used the snowballing technique. Data were collected via in‐depth interviews. The recorded data were transcribed and coded into themes and subthemes via thematic analysis.

**Results:**

The study found that two main attributes were common among NCD patients living with complications, namely, a lack of access to sanitation, including poor design of sanitation, loss of dignity, and lack of education on sanitation. They also faced challenges accessing health care, including long waiting times and a shortage of staff.

**Conclusion:**

In conclusion, the study confirms that people living with NCD complications in resource‐limited settings require special care to improve quality of life and dignity.

## Graphical abstract

The study investigates the experiences of noncommunicable disease (NCD) patients living with complications in Mzuzu, Malawi, focusing on sanitation issues. Through in‐depth interviews with nine individuals, it reveals a lack of modern facilities and sanitation education. The conclusion emphasizes the need for specialized care to enhance the quality of life and dignity for NCD patients in resource‐limited settings.

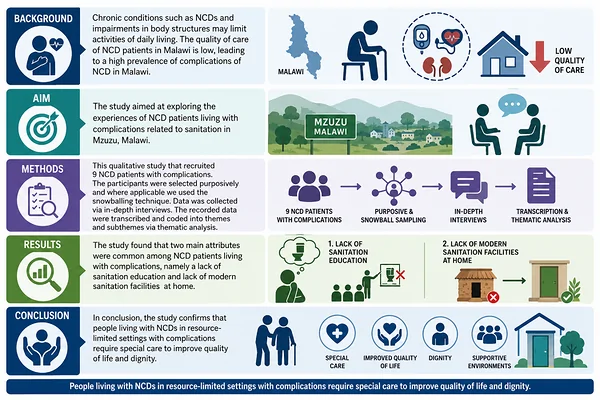

## 1 Introduction

Globally, noncommunicable diseases (NCD) account for 71% of deaths [1]. Historically, the burden of disease in sub‐Saharan Africa (SSA) has been dominated by infectious conditions; however, as of late, the region has experienced an upsurge in the burden of NCDs [2]. Currently, the SSA region faces multiple NCD conditions, including cardiovascular conditions, mental health, neoplasms, digestive disorders, and some cancers. Among all, the top four killers include cardiovascular diseases, respiratory diseases, diabetes, and cancers [1]. In particular, the SSA region is vulnerable to the development of multiple chronic conditions due to high rates of poverty and poor health education and health service delivery systems.

Apart from death, NCDs such as diabetes have the potential to lead to complications due to their chronic and insidious nature [3]. For example, complications of diabetes include nephropathy, neuropathy, retinopathy, and macrovascular complications. In addition, an untreated diabetic condition can result in complications such as foot ulcers, kidney failure, and stroke [4]. Specifically, a foot ulcer refers to a group of symptoms related to ischemia, neuropathy, and infections that may sometimes lead to amputation [5]. Overall, NCDs are chronic conditions that could lead to impairments, limitations in functionality, restrictions in participation, reduced quality of life, and compromised mental health [6].

In Malawi, the prevalence of diabetes was recorded at 3% in urban areas and about 1% in rural areas, with diabetes common among 14% of the urban population compared to 13% of the rural population [7]. In another study conducted among Malawians, the prevalence of diabetes was 5% [5]. Needless to say, Malawi continues to grapple with hypertension and diabetes in the face of limited resources, leading to poor quality of care and eventual impairments. For instance, a study conducted in 55 health centers found that 38% of health centers have glucometers and 24% have glucose dipsticks. In addition, the study found that first‐line medication for the treatment of diabetes was available in 4% of the health centers [7]. Not surprisingly, the disease burden for NCDs in Malawi remains high, recorded at 31% measured in disability‐adjusted life years [8].

NCDs are among the leading causes of disability [9]. Disability from a prolonged health issue can lead to disturbance in the performance of activities of daily life, including work [10]. Accordingly, disability is mainly characterized by physical disabilities and how it interacts with environmental characteristics as well as individual behavioral hindrances that prevent equal participation with others in society [11]. As a consequence, physical disability can negatively affect the quality of life and eventually lead to an increase in healthcare costs [10]. The incurable nature of disabilities means that there is a need for ongoing self‐care management by the individual. When self‐care management is incorporated for an individual in alignment with their demands, life quality and work productivity improve. Nevertheless, the World Health Report on equity noted that functional limitations can cause struggle when accessing specialized services [12]. Furthermore, a study conducted in Indonesia found that less than 5% of households had inclusive sanitation facilities, including supportive tools and bins, attributable to low participation by people with disabilities in decision‐making regarding sanitation programmes [11].

When people with NCDs practise poor sanitation, it can lead to medical issues, such as diarrheal disease, malnutrition, and neglected tropical diseases [11]. However, a study conducted in Malawi found that body function limitations, such as incontinence, pain, and inability to communicate WASH needs, were some of the significant barriers to accessing WASH needs [13]. Another study conducted in a diabetic clinic in Blantyre found high prevalence rates of complications among diabetes patients such that approximately 34.7% had retinopathy, 46.4% had neuropathy, and about 34.7% developed nephropathy [14]. Similarly, the findings of the diabetic retinopathy study, which included 357 patients with diabetes, found that 14.5% of the participants had neuropathy while 2.2% had amputations [5]. Additionally, the prevalence of diabetic retinopathy among diabetic patients was reported as high as 50.1%, which is three times more than what is usually reported among European populations [15].

In Nigeria and neighboring Zambia, studies have reported on the experiences of people with disabilities [16, 17]. Previous studies conducted in Malawi have documented the experiences of patients with chronic conditions [6] and the epidemiology of NCD and their complications [5, 14, 15]. Others documented the experiences of people with disabilities when accessing WASH facilities in Malawi; nevertheless, the study included participants with many causes of impairments [13]. Moreover, a recent study investigated the experiences of NCD patients accessing care but neglected their experiences at home, which could also influence health and care‐seeking behavior [18]. Nevertheless, to the best of our knowledge, there is a dearth of knowledge on the experiences of people living with NCD complications in Malawi. Therefore, the study aimed to explore the experiences of people living with NCD complications and focuses on access to health and sanitation facilities. The work is embedded in the social model of disability [13]. The model classifies the challenges faced by people with disabilities into three distinct categories, including physical barriers, institutional barriers, and social barriers. The findings of the study will help planners, managers, and policymakers formulate policies and strategies to ensure optimal care and well‐being for NCD patients living with complications in Malawi.

## 2 Methods

### 2.1 Study Site

The study was conducted at Ekwendeni in Mzuzu, Northern Malawi. The population is predominantly Ngoni.

### 2.2 Study Design

The study employed a qualitative study design to explore the experiences of NCD patients living with complications.

### 2.3 Study Population

The study population comprised NCD patients living with complications.

### 2.4 Inclusion Criteria

The study included participants aged 15–85: NCD patients living with complications, with a focus on those with physical impairments.

### 2.5 Exclusion Criteria

The study excluded NCD patients living with complications outside the Ekwendeni catchment area.

### 2.6 Sampling Techniques

The study used a purposive sampling method as stipulated in the WHO framework, which uses disease registers. Furthermore, the study used a snowball method of sampling to reach patients who might have been missed by the hospital registration.

### 2.7 Sample Size

A total of nine NCD patients living with complications were recruited for the study on the basis of data saturation. None of the participants approached to participate, refused, or withdrew from the study.

### 2.8 Data Collection Methods

The study data collection tool initially went through pilot testing, and based on the findings, the tool was revised. Primary data was collected through open‐ended questionnaires in the form of interview guides, which included prompts to allow participants to provide further explanation. The face‐to‐face interviews were conducted by the lead author in a private and confidential setting convenient for the participants within their respective homes. No other person apart from the interviewer and interviewee was present during data collection. The interviewer, who is also the lead author (female), is a PhD candidate and an experienced public health practitioner with a special focus on sanitation, currently working as director of research at Livingstonia University in Malawi. To avoid introducing bias and allow for objectivity, regular debriefings were conducted to enhance transparency. The interviews were audio‐recorded and lasted about 30–40 min. The data collection happened between February and March 2022. We used the Consolidated Criteria for Reporting Qualitative Research (COREQ) 32‐item checklist (Supporting Information).

### 2.9 Data Analysis

Qualitative data were prepared for analysis in three main ways, including translating to English and verbatim transcripts (written record of an IDI). Transcribing: word‐for‐word. The transcripts were familiarized, then they were coded to identify meaningful segments according to study objectives following comparisons across transcripts. Next, conceptually related categories were examined and grouped into subthemes, which, in turn, were also reorganized into overarching themes. Moreover, the research applied the thematic analysis approach. Two coders manually coded the data themes. Themes were identified from the data. The study used deductive reasoning by establishing themes a priori. Two researchers independently generated the codes and reached a consensus through reflexive conversation. Where two could not resolve, a third researcher was involved. The participants were not allowed to provide feedback on the transcripts since transcribing was conducted by two researchers separately and an agreement was reached by involving a third researcher whenever disagreements arose.

### 2.10 Ethical Considerations

The study was approved on January 29, 2022, by the Mzuzu University Research Ethics Committee (ref No: MZUNIREC/DOR/26/18). Permission was granted by the Mzimba North District Health Office, hospital management, and village elder/headman in the specific villages where data was collected. The study followed the Declaration of Helsinki and included confidentiality and not pressuring participants to speak and ensured anonymity by removing all identifiers in the data. In addition, the study obtained and used informed consent.

## 3 Results

The study identified two main themes and five subthemes. The main themes include challenges related to accessing sanitation services and health system barriers to accessing care among patients living with NCD complications (Table 1).

**TABLE 1:** Themes and subthemes.

**Theme 1**	Challenges faced when accessing sanitation facilities
Subtheme 1:1	Poor design of sanitation facilities
Subtheme 1:2	Loss of dignity when accessing sanitation facilities
Subtheme 1:3	Lack of sanitation education
**Theme 2**	Health system barriers to accessing care
Subtheme 2:1	Long waiting time
Subtheme 2:2	Shortage of staff

### 3.1 Challenges Faced When Accessing Sanitation Facilities

#### 3.1.1 Poor Design of Sanitation Facilities at Home

Participants explained that access to sanitation facilities and services was a hurdle due to mobility challenges and the types of sanitation facilities in use in the homes because they were not user‐friendly for their conditions.

A diabetic patient shared his experience concerning the location of a toilet and how to get to the toilet when mobility is compromised,

As you can see, my toilet is outside the house. When I was walking properly, even though I had lost my leg, I could still access the toilet. Now that I am losing my sight and I can't see properly, P1

Another NCD patient complained about the usability of the facility and how they are constructed;

I will say two things. It is all about the design of the toilets we have; they are not easy to use, P2

#### 3.1.2 Loss of Dignity When Accessing Sanitation at Home

In comparison, patients said sanitation facilities used at the hospital were better, and the guardians were given bedpans to use, though patients were not comfortable using them. At home, they used either rolled grass or old dirty clothes or old tins instead of bedpans, which they did not have and could not afford to buy. Use of existing sanitation facilities at home leads to diminished self‐respect and dignity of the patient living with NCD complications when accessing the sanitation facilities at home.

A patient said,

At the hospital we used bed pans, but here it is terrible sitting on rough grass, on old dirty clothes, or just where you are lying as if you are a child; my dignity is lost!!, Most of the time feel like not eating at all, not to bother my guardians mmmhuuuu!!!, Maybe better at the hospital when admitted and using a bedpan, people were asked to go out. Respect could be there but here at home, noooooo!!!, it is terrible!! Of course, we cannot afford to buy a bedpan; they are expensive. They are the same guardians, but in the hospital, the beddings were changed every day, not here!! P2

Another participant complained that living with NCD complication has led to loss of independence and having to rely on otherwise, he risks harming himself when he tries to access sanitation facilities on his own.

I have to drag myself on the ground, and sometimes thorns prick me in the process, and I develop wounds. Now I am having a hard time going to the toilet on my own; I have to depend on my wife to help me, which cannot always happen, as she also has to do some other tasks to help provide for the home now that I cannot. Besides, I don't like to ask for such help as it is not fair to the person helping. And for me, where is my dignity? P1

The sanitation facilities in most patients’ homes required assistance to be used by those who were blind or unable to walk independently (mobility challenges). Again, patients felt bad for the person handling their waste products. One patient said,

it is difficult for us to have movable toilets as they have at the hospital because it will mean that someone will have to go and throw the waste away for us, and that is not fair to them. But I would recommend the one I saw this one time when I was working in Chitipa. The toilet had a seat on it, and with that, it is easy to use, and you can easily sit and get up without many problems. P 3

#### 3.1.3 Lack of Sanitation Education for NCD Patients With Complication

The participants repeatedly mentioned that they got health education on feeding, compliance to medication, and maybe bathing (hygiene). No education in relation to sanitation. They kept recommending that sanitation education in NCD was very important and needed, although it was lacking from healthcare workers.

A hypertensive patient said,

It is important to learn how to care for the patient and then learn the plans to do and not just to worry about preventing the BP, but also look after your patient well. Our toilets are difficult to use when one is in this condition. P5

Another patient commented that,

We have no idea what we are doing sometimes, since we have not been taught. We had this other Health Surveillance Assistant who used to come and help us with sanitation issues, but now we have forgotten. It's been years since we last saw a health worker here telling us about sanitation. It would be really good to learn because now we just do what we feel is okay, but you, from the hospital, are the ones who know, so you have to teach us. We do open defecation in our houses, how do you go to the toilet when you do not walk, worse at night…… P6

### 3.2 Health System Barriers to Accessing Care

#### 3.2.1 Long Waiting Time

The study identified that there were health system bottlenecks that also affected patients with NCD, such as long waiting hours, which affected the quality of care received by patients with complications. One participant complained that getting to the hospital was not a challenge, but the challenge was long waiting hours before accessing care.

Since I started going to the central hospital, they received us well and didn't show any attitude. Even here at Ekwendeni hospital, there was no problem. Only once it is when I was not welcomed by either a clinician or doctor, I don't know who it was. Granny got sick during the night and I rushed to the hospital to call for an ambulance to come get us. The driver was not a problem, the ambulance took us to the hospital, there a problem came up; we were detained for three hours unattended to. P5

#### 3.2.2 Shortage of Staff

Some patients were not receiving care when they went for their review because of shortage of staff. There was only one nurse responsible for the NCD clinic; when she was not available, patients were not reviewed; instead, they were being given another review date to wait for the return of the NCD nurse. A diabetic patient explained his experience on follow‐up hospital visits after discharged on home‐based care.

He said,

Most of the time they treat us well. I can't say that they don't help. However, there is the nurse that normally attends to us who is in palliative and NCD patient care. The problem is when we go to the hospital and this nurse is not there. Other nurses and doctors say that your nurse is not here so wait or come on the day that she will be available. And just like that we sometimes don't get help because she is not around. P4

## 4 Discussion

The study found that patients living with NCD complications experience challenges accessing sanitation facilities, including poor design of sanitation facilities, loss of dignity, and lack of sanitation education. Other challenges emerging from the study were related to accessing healthcare services, such as long waiting times and staff shortages. Nevertheless, the study has shown that the challenges are not solely due to the impairment resulting from the NCD complications but rather from the contextual factors. The study aligns well with the social model of disability, which postulates that people with physical impairments oftentimes face challenges not as a result of the impairment but restrictions in the physical, social, and institutional barriers. The findings of this study highlight the need to incorporate management strategies beyond clinical practice to address challenges in accessing sanitation and health services for people living with impairments related to NCD complications in the area. These issues are critical to ensure the livelihood of persons with NCD complications to enjoy a good quality of life and dignity.

In this study, it was found that patients with NCD complications struggle to access sanitation facilities, mainly due to the poor design of sanitation facilities at their homes. Similarly, a study conducted in neighboring Zambia found that the traditional toilets are designed poorly without considering the buy‐in from people with disabilities [17]. For instance, the study reported that traditional designs did not have wide enough doors to allow people with disabilities to enter using a wheelchair. Another study conducted in Nigeria observed that toilets had no ropes or handrails to aid usage by people with disabilities [16]. The findings of our study can be attributed to locomotion, which is limited in patients with activity‐limiting impairments. Traditionally, sanitation in Malawi mainly comprises a hole in the ground surrounded by a wall made of bricks or grass [19]. This finding aligns well with the social model of disability, which postulates that NCD complications and structural designs interact to limit activities of daily living, making it difficult to live independently, including practicing good sanitation in low‐resource settings with poor social support structures [6]. The findings of our study suggest that people with NCD complications face challenges beyond the clinical management of NCD, calling for disability‐inclusive NCD care considering functional limitations and accessibility requirements. Moreover, it highlights the need to plan and design disability user‐friendly sanitation services to ensure accessibility by people living with NCD complications. Specifically, the study recommends building disability‐friendly sanitation facilities that align with universal design principles and accessibility guidelines [20].

The study found that patients living with complications were suffering from loss of dignity when accessing sanitation facilities. The participants cited loss of independence when using sanitation facilities, whereby they have to rely on help from another person. The participant's reliance on a caregiver meant more than a practical coping mechanism. Where environmental barriers prevented patients from conducting daily activities independently, caregivers became intermediaries between patients and the environment, questioning the autonomy of the patient living with NCD complications. This finding is similar to a report from Vanuatu, which pointed out that before installing sanitation‐friendly facilities, people with disabilities were reliant upon someone for guidance and assistance [20]. The finding of our study can be explained by the poor design of sanitation facilities alluded to earlier. The findings of our study suggest the need for planners to move the focus from the provision of sanitation facilities to consider issues such as usability to promote independent sanitation for people with functional limitations, such as NCD patients with complications.

The study reported that patients with NCD complications lack education on sanitation as it relates to activities of daily living and self‐management. This can emanate from a lack of social support structures in low‐resource settings such as Malawi [6]. This finding is not surprising considering that training in sanitation in Malawi is currently compartmentalized within engineering and environmental practitioners as the main technical disciplines [19]. Furthermore, the findings of the study can be explained by a lack of knowledge and skills related to sanitation education among healthcare workers, as reported among nurses caring for NCD patients with amputations in Ghana [21]. This highlights the need for in‐service training for healthcare workers and social workers to ensure they can disseminate sanitation education to this vulnerable population and reduce the risk of infection when accessing sanitation services. This finding suggests the need to incorporate sanitation education among other professionals such as social workers and healthcare workers. Specifically, there is a need to consider using community health workers in improving sanitation education among patients with NCD complications. Overall, this could help ensure that people living with NCD complications can maintain a positive self‐worth and dignity to enjoy a happy and fulfilling life.

The current study found that people living with NCD complications faced challenges accessing healthcare services and cited long waiting times. Long waiting times have been widely cited across the Malawian healthcare literature [22]. The findings of our study can be explained by the shortage of staff. The shortage of healthcare professionals, such as nurses and clinicians, is a well‐known barrier to quality of care amid decentralization in Malawi [8]. The study recommends the integration of services through an integrated care model such as PEN Plus, which is recommended in areas where human resources are scarce and allows for NCD care at intermediate levels. Moreover, the study implies that there is a need to ensure health system responsiveness to meet the needs of people with NCD in the context of disability to ensure that barriers experienced by vulnerable groups are addressed and not rely on standard service arrangement that may not promote equal access for all.

### 4.1 Limitations

The study is not without limitations. First, we could not ascertain the association between dependent and independent variables. Second, the study findings are constrained to the setting of the study and cannot be generalized to the entire population. Moreover, data regarding age, sex, and NCD diagnosis, including physical impairment or complication, was not captured. Furthermore, the study lacks reliability due to the absence of member checking of results. In addition, more limitations relate to the smaller sample size as well as use of purposive and snowballing methods of selecting participants, which could lead to bias. Regardless, this study is the first of its kind to unravel a novel topic within the study population.

## 5 Conclusion

In conclusion, the study found that people living with NCD complications are facing challenges related to accessing sanitation services, including a lack of sanitation education, poor design of sanitation facilities, and loss of human dignity when accessing sanitation facilities. Moreover, the study found that when accessing care, people living with NCD complications often face long waiting times and a shortage of staff. The findings of the study highlight the need to consider sanitation‐friendly facilities for patients living with NCD complications in the study area by ensuring sanitation design improvements and revamping sanitation education targeting the patients, healthcare workers, and caregivers in order to improve human dignity and respect for patients living with NCD complications in the area. Furthermore, the study recommends the eradication of barriers to accessing care to ensure easy access to health facilities by patients living with NCD complications. Further studies can employ a quantitative design to establish relationships among variables and quantify the magnitude of the phenomenon.

## Author Contributions

**Mary Kumwanje**: conceptualization, investigation, writing – original draft, writing – review and editing, visualization, methodology, formal analysis, data curation, supervision, project administration. **Thokozani Mzumara**: writing – original draft, writing – review and editing, validation, data curation. **Isaac Ndemera**: investigation, writing – original draft, writing – review and editing, methodology, formal analysis.

## Funding

The authors have nothing to report.

## Ethics Statement

The study was ethically approved by the Mzuzu University research committees.

## Consent

The study through M.K. obtained written informed consent from all participants between February 17 and March 18, 2022.

## Conflicts of Interest

The authors declare no conflicts of interest.

## Permission to Reproduce Material From Other Sources

Not applicable.

## Supporting information




**Supporting File**: puh270402‐sup‐0001‐SuppMat.pdf

## Data Availability

The data are available upon a reasonable request.

## References

[1] J. J. Bigna and J. J. Noubiap , “The Rising Burden of Non‐Communicable Diseases in Sub‐Saharan Africa,” Lancet Global Health 7, no. 10 (2019): e1295–e1296, 10.1016/S2214-109X(19)30370-5. 31537347

[2] H. N. Gouda , F. Charlson , K. Sorsdahl , et al., “Burden of Non‐Communicable Diseases in Sub‐Saharan Africa, 1990–2017: Results From the Global Burden of Disease Study 2017,” Lancet Global Health 7, no. 10 (2019): e1375–e1387, 10.1016/S2214-109X(19)30374-2. 31537368

[3] K. Papatheodorou , M. Banach , M. Edmonds , N. Papanas , and D. Papazoglou , “Complications of Diabetes,” Journal of Diabetes Research 2015 (2015): 189525, 10.1155/2015/189525. PMC4515299 26247036

[4] R. Balaji , R. Duraisamy , and M. P. Kumar , “Complications of Diabetes Mellitus: A Review,” Drug Invention Today 12, no. 1 (2019): 98, https://openurl.ebsco.com/EPDB%3Agcd%3A6%3A29809558/detailv2?sid=ebsco%3Aplink%3Acrawler‐gcd&id=ebsco%3Agcd%3A134240957&crl=c&jrnl=09757619&link_origin=scholar.google.com.

[5] M. M. Kasiya , G. D. Mang'anda , S. Heyes , et al., “The Challenge of Diabetic Foot Care: Review of the Literature and Experience at Queen Elizabeth Central Hospital in Blantyre, Malawi,” Malawi Medical Journal 29, no. 2 (2017): 218–223, 10.4314/mmj.v29i2.26. PMC5610299 28955436

[6] E. Samwiri Nkambule and G. Msiska , “Chronic Illness Experience in the Context of Resource‐Limited Settings: A Concept Analysis,” International Journal of Qualitative Studies on Health and Well‐Being 19, no. 1 (2024): 2378912, 10.1080/17482631.2024.2378912. PMC11251436 39007854

[7] C. Pfaff , G. Malamula , G. Kamowatimwa , et al., “Decentralising Diabetes Care From Hospitals to Primary Health Care Centres in Malawi,” Malawi Medical Journal 33, no. 3 (2021): 159–168, 10.4314/mmj.v33i3.3. PMC8843181 35233273

[8] T. Ruderman , E. Chibwe , C. Boudreaux , et al., “Training Mid‐Level Providers to Treat Severe Non‐Communicable Diseases in Neno, Malawi Through PEN‐Plus Strategies,” Annals of Global Health 88, no. 1 (2022): 69, 10.5334/aogh.3750. PMC9389951 36062045

[9] B. Khandelwal and C. Gupta , “Leading Causes of Death and Disability Among the Global Aging Community,” in The Ageing Population, ed. P. Lama (Springer, 2023), 37–54, 10.1007/978-981-99-5772-9_2.

[10] D. W. Maidment , K. Clarkson , E. V. Shiel , K. Nielsen , J. Yarker , and F. Munir , “A Rapid Systematic Review Assessing the Effectiveness of Interventions to Promote Self‐Management in Workers With Long‐Term Health Conditions and Disabilities,” International Journal of Environmental Research and Public Health 21, no. 12 (2024): 1714, 10.3390/ijerph21121714. PMC11728185 39767553

[11] D. Daniel , A. Nastiti , H. Y. Surbakti , and N. M. U. Dwipayanti , “Access to Inclusive Sanitation and Participation in Sanitation Programs for People With Disabilities in Indonesia,” Scientific Reports 13, no. 1 (2023): 4310, 10.1038/s41598-023-30586-z. PMC10017721 36922602

[12] R. G. Lanzi , R. A. Modi , and J. Rimmer , “A Disability‐Inclusive Healthcare‐to‐Well‐Being Translational Science Framework,” International Journal of Environmental Research and Public Health 21, no. 1 (2023): 18, 10.3390/ijerph21010018. PMC10815666 38248483

[13] S. White , H. Kuper , A. Itimu‐Phiri , R. Holm , and A. Biran , “A Qualitative Study of Barriers to Accessing Water, Sanitation and Hygiene for Disabled People in Malawi,” PLoS ONE 11, no. 5 (2016): e0155043, 10.1371/journal.pone.0155043. PMC4865162 27171520

[14] D. B. Cohen , T. J. Allain , S. Glover , et al., “A Survey of the Management, Control, and Complications of Diabetes Mellitus in Patients Attending a Diabetes Clinic in Blantyre, Malawi, an Area of High HIV Prevalence,” American Society of Tropical Medicine and Hygiene 83, no. 3 (2010): 575–581, 10.4269/ajtmh.2010.10-0104.

[15] P. I. Burgess , “Determinants of Severity and Progression of Diabetic Retinopathy in Southern Malawi” (Doctoral diss., University of Liverpool Repository, 2015), 10.17638/02033719.

[16] E. O. Oloruntoba , I. P. Udofia , and M. Adejumo , “Access to Sanitation Facilities and Handwashing Practices Among Physically Challenged Persons in Homes for the Disabled in Ibadan, Nigeria,” Journal of Environmental Protection 11, no. 4 (2020): 299–314, 10.4236/jep.2020.114017.

[17] J. Mwale , V. Kanyamuna , J. Moose , and F. Simui , “Disability Friendly Sanitation Facilities in George Compound in Lusaka, Zambia: A Stakeholders Perspective,” Journal of Arts, Humanities and Social Science 3, no. 1 (2026): 76–83, 10.69739/jahss.v3i1.1429.

[18] C. Boudreaux , M. Mansoor , M. B. Aron , et al., “Pathways to Care for Patients With Severe Chronic NCDs: A Cross‐Sectional Survey of Inpatients in Neno Malawi,” Tropical Medicine & International Health (2026), 10.1111/tmi.70211.42526854

[19] B. A. Chunga , D. Mkwambisi , C. L. Workman , F. L. de Los Reyes , and R. H. Holm , “Educating Sanitation Professionals: Moving From STEM to Specialist Training in Higher Education in Malawi,” Waterlines 41, no. 3 (2022): 1–13, 10.3362/1756-3488.22-00662.

[20] A. Coleman , Making Inclusion Meaningful: The Human Centred Design Approach to Building Latrines for People With Disabilities in Vanuatu (Institute of Development Studies and Partner Organisations, 2024), 10.19088/SLH.2024.006.

[21] B. E. Atinga , A. Henyo , C. A. Ayamga , T. Perekuu , and R. Braimah , “Exploring Nurses' Experiences and Challenges in Managing Diabetic Amputations: A Qualitative Study,” BMC Nursing 24, no. 1 (2025): 499, 10.1186/s12912-025-03143-4. PMC12060339 40340700

[22] V. Shiroya , N. Shawa , B. Matanje , et al., “Reorienting Primary Health Care Services for Non‐Communicable Diseases: A Comparative Preparedness Assessment of Two Healthcare Networks in Malawi and Zambia,” International Journal of Environmental Research and Public Health 18, no. 9 (2021): 5044, 10.3390/ijerph18095044. PMC8126199 34068818

